# A Survey of Current Tobacco and Nicotine Product Users to Identify Barriers to Quitting Smoking in Germany

**DOI:** 10.7759/cureus.82419

**Published:** 2025-04-17

**Authors:** Christoph Neubert, Nelson Tewes, Alexander K Nussbaum

**Affiliations:** 1 Scientific and Medical Affairs, Philip Morris GmbH, Gräfelfing, DEU

**Keywords:** barriers to quitting smoking, e-cigarettes, heated tobacco products, smoke-free products, smoking, tobacco, tobacco harm reduction

## Abstract

Background: Smoking rates remain high in Germany despite the well-known serious health risks. This survey assessed motivation to stop smoking in Germany and barriers to quitting smoking or switching to two types of smoke-free products (SFPs): e-cigarettes (E-cigs) and heated tobacco products (HTPs).

Methods: In 2022, computer-assisted web interviews were conducted among adults (>19 years) who smoked (n=1,000) or used SFPs (n=196).

Results: Overall, 51.2% of people who smoke were not motivated to stop smoking, 29.1% reported an intention to quit in the following year, and the remainder had some desire to quit but had not decided when to try. Lack of motivation was highest among people who smoke aged >65 years (64.4%) and lowest among those aged 19-34 years (38.9%). People with lower socioeconomic status who smoked had less motivation to quit than those with a higher socioeconomic status. In the sample of people who smoke, the barriers to quitting smoking included enjoyment of smoking (50.1%), difficulty in changing habits (41.4%), and lack of discipline (31.2%), with key differences among age groups. Only 27.3% of people who smoke perceived E-cigs/HTPs to have lower health risks relative to cigarettes, versus 84.7% of SFP users. Among SFP users, 41.8% reported a period of dual use when switching away from cigarettes. For the vast majority (89.1%), this period lasted less than a year, and 96.3% reported reduced cigarette consumption during this phase.

Conclusions: Despite the well-known health risks, a large number of people in Germany continue to smoke, with many not motivated to quit. The primary barrier to quitting is the enjoyment of smoking. Additionally, there are misperceptions about the relative risks of cigarettes versus SFPs among people who smoke. To effectively reduce smoking prevalence, targeted campaigns must address these barriers and correct these misperceptions. Existing tobacco control strategies should be supplemented with tobacco harm reduction approaches to drive down smoking prevalence as quickly as possible.

## Introduction

The German Study on Tobacco Use (DEBRA) reported that around 30% of the population (~20 million people) smoked cigarettes in 2024 [1]. The serious health risks of smoking cigarettes are well known, yet in Germany alone, an estimated 127,000 premature smoking-related deaths occur each year [2]. Despite public health campaigns to encourage cessation, smoking prevalence has not declined in the last seven years [1]. This is unsurprising given that the majority of people who smoke in Germany no longer engage in serious quit attempts, with less than 10% of people who smoke having made a serious cessation attempt in the previous 12 months [1,3]. This is alarming since many people who smoke need 30 or more quit attempts before they are successful, and only 3%-6% of those who try to quit achieve long-term abstinence [4,5].

Although tobacco control methods, including smoking and advertising bans and increased taxation in the early 2000s, led to a decline in smoking prevalence, that decline has now plateaued [1,6]. New approaches are needed to motivate people who smoke to quit. The S3 Guideline for Smoking and Tobacco Dependence recommends behavioral therapy combined with pharmacotherapy (e.g., nicotine replacement therapy (NRT), varenicline, bupropion, and cytisine) [7]. Other complementary approaches, e.g. tobacco harm reduction, could help support people who smoke and lack cessation motivation to move away from cigarettes [8-11].

Regulatory bodies and governmental agencies acknowledge that most health risks associated with smoking arise from the inhalation of harmful and potentially harmful constituents (HPHCs) generated by tobacco combustion [12]. Although nicotine is addictive, it is not carcinogenic in itself, and it is not the primary cause of smoking-related diseases [9,13-15]. This has led to the concept of “tobacco harm reduction”, whereby people who smoke are encouraged to switch from smoking cigarettes to using smoke-free products (SFPs) that deliver nicotine without combustion, such as e-cigarettes (E-cigs) or heated tobacco products (HTPs) [16,17]. Although SFPs contain nicotine, which is addictive and not risk-free, they emit substantially reduced levels of HPHCs linked to the development of smoking-related diseases [9,18-20]. If enough people who smoke and do not quit switch to SFPs, whose use is associated with lower HPHC exposure compared to smoking, it may be possible to dramatically reduce the public health impacts of smoking-related disease [9,15,19,21].

Switching to SFPs requires that adults who smoke have access to appropriate information, which is a key aspect of making informed consumer decisions. However, there are widespread misconceptions about the relative harms of SFPs and nicotine, even among physicians. Surveys from several European countries and the United States (US) revealed that many physicians wrongly believe that nicotine directly causes smoking-related diseases [22-26]. Furthermore, German media reporting about the relative harms of SFPs and nicotine are sending inconsistent messages. Misperceptions about the relative harm of nicotine and alternative products might be one of the reasons why relatively few people who smoke in Germany have adopted SFPs [20,27].

We previously investigated whether adults who smoke in Germany are motivated to quit and asked what barriers prevent them from doing so. In our previous survey of 1,000 people who smoke, 54% were not motivated to stop smoking cigarettes, while only 29% intended to quit at some point [28]. To build on that work, motivation to quit smoking was explored by identifying (1) the types of barriers that impede people who smoke from quitting and (2) the perceptions of the harms of cigarettes relative to SFPs. We compared the responses of people who currently smoke with those of former exclusive smokers who have largely switched to SFPs. The results pinpoint a specific knowledge gap in health risk perceptions and motivation to quit.

Selected results from this article were previously presented as an oral presentation at the Global Forum on Nicotine (21-24 June 2023) and as a poster at the “Interdisziplinaerer Suchtkongress” (30 June-02 July 2023).

## Materials and methods

Study design

A cross-sectional, observational, web-based study was used to obtain data on tobacco and nicotine product (TNP) use and motivation to quit smoking. The study was conducted in Germany among people who smoke and SFP users between October and November 2022. NielsenIQ/GfK (Nuremberg, Germany) carried out the fieldwork and supervised the study.

Ethical approval was not required for this anonymized opinion survey because it was non-interventional and did not pose a risk to the participants. The study conforms with the World Medical Association’s International Code of Medical Ethics (Declaration of Helsinki) and applicable regulatory requirements. The survey was administered by NielsenIQ/GfK in accordance with their quality system. NielsenIQ/GfK complies with the International Chamber of Commerce/European Society for Opinion and Market Research (ICC/ESOMAR) International Code on Market, Opinion and Social Research, and Data Analytics.

All participants gave their consent, including the use of their anonymized data in publications, and received compensation for their time in the form of loyalty points, which can be redeemed online for various rewards, such as physical gifts, online vouchers, donations to charities, or bank transfers.

Study population

The study population was recruited by email invitation from one of two online market research panels (Bilendi, Berlin, Germany or Cint, Berlin, Germany) based on the inclusion criteria below. The sample was selected to be representative of the adult smoking population in Germany by setting quotas based on previously reported characteristics including sex, age, and German state [28,29].

The inclusion criteria were: 19 years of age or older, smoking at least one cigarette per day, and no use of SFPs (for the population who smoke), or had previously smoked cigarettes daily or occasionally, and had now switched to using an SFP at least 15 days per month (for the population who switched to SFPs). The exclusion criteria were pregnancy and breastfeeding; affiliation with the tobacco industry or its retail businesses; and employment in the fields of market research, public relations, or journalism.

To ensure a clear distinction between the groups of people who smoke and people who switched to SFPs, people who initiated with SFPs were excluded, and, initially, dual users were excluded as well. However, to enhance the recruitment rate without compromising the study’s integrity, this criterion was adjusted. Rare (smoking cigarettes three to four times per month) and occasional (smoking cigarettes five to 14 times per month) dual users were included in the SFP sample, while dual users, who used cigarettes often (smoking cigarettes 15-29 times per month) remained excluded. This allowed for further comparisons within the group of users fully or mostly switching to SFPs.

At present, there is no standardized definition for “dual use.” The US Centers for Disease Control defines dual use as when “some people try to cut back on smoking cigarettes or work toward quitting smoking completely by using e-cigs, smokeless tobacco, or other tobacco products in addition to regular cigarettes,” which is very different from a United Kingdom (UK) definition of “the use of E-cigs alongside cigarettes outside of a quit attempt” [30,31]. For the purposes of this study, dual use is defined as the concurrent use of cigarettes and SFPs.

Study survey

The survey was designed to gain insights into motivation to quit smoking and barriers to quitting in Germany among adults who smoke. It was administered via computer-assisted web interviewing. Data were collected on sociodemographic, socioeconomic, TNP use, motivation to quit, and perceptions of the relative risks of TNPs.

The DEBRA study (Heinrich Heine University of Duesseldorf) assesses TNP use and motivation to quit smoking. The survey questions were designed to build on the DEBRA study by more closely examining motivation to quit and explicitly exploring barriers to quitting (see below for modifications to DEBRA). The survey was accessed online via a computer, smartphone, or tablet and was designed to take less than 15 minutes to complete.

Sociodemographic Data

Participants were asked to provide their age (in years); sex; pregnancy status (females); level of education attained (elementary school with or without apprenticeship, high school with or without diploma, some college but no degree, and college with degree); employment status (student, housewife/husband, retired, part-time, full time, unemployed, and no answer); and household net monthly income.

TNP Use, Motivation to Quit, and Quit Attempts

For all participants, data were collected on TNP use, previous quit attempts, perceived barriers to quitting, perceptions of the relative risks of using different TNPs, and whether physicians ever mentioned quitting to them. In addition, participants were asked why they had not yet tried E-cigs/HTPs, and SFP users were asked about their dual use of cigarettes and E-cigs/HTPs during the transition period from smoking to SFP use along with motivation to quit smoking.

Motivation to Stop Smoking

To assess motivation to quit smoking, a modified version of the “Motivation to Stop Scale” (MTSS) was used [32,33]. The adaption omitted question 5 (“I want to quit smoking cigarettes and hope to do so in the near future”) and added two additional questions (“I definitely want to quit and aim to within the next 12 months” and “I definitely want to quit and aim to within the next six months”). Additional covariates were captured relating to cigarette smoking habits and quit attempts and correlated with sociodemographic and socioeconomic characteristics.

To investigate why people who smoke continue to smoke cigarettes, all participants were asked both a closed question and an open question, thus giving a more holistic view of the study population. The closed question (“From your point of view, what are crucial obstacles and difficulties that prevent you from quitting smoking cigarettes?”) allowed a fixed choice of one or more of 17 possible responses, thus enabling quantification. The responses were subsequently grouped into five categories (capability, opportunity, attitude, social support, and self-efficacy). The closed question was asked to all participants, with the SFP users being asked to answer with respect to how they felt before adopting SFPs. In addition, an open question was added to give insights into responses that were not anticipated.

Study objectives

The main objectives of this observational study were (1) to assess overall motivation to quit smoking in Germany and identify associated sociodemographic and socioeconomic factors; (2) to determine the barriers to quitting smoking; (3) to understand the reasons of people who smoke for not switching to SFPs and their risk perceptions of these alternative products; and (4) with respect to the specific sample of SFP users who are former exclusive smokers, to estimate the extent of dual use during the transition period.

Data analysis

No formal sample size calculation was performed; however, the aim was to recruit 1,000 adults who smoke and 200 adults who use SFPs and had previously smoked. By comparison, each DEBRA wave surveys 2,000 Germans aged 14+, corresponding to ~600-7,700 people who smoke. As this was an observational study, data are reported as simple frequencies.

## Results

Study population

The study recruited a total of 1,196 participants from across Germany, comprised of 1,000 adults who smoked at least one cigarette per day and did not use SFPs and 196 adults who use SFPs (127 (64.8%) E-cig users and 69 (35.2%) HTP users). The sociodemographic data are summarized in Table 1. The mean ages of both groups were similar (people who smoke: 47.3 years; SFP users: 45.1 years), as was their highest level of educational attainment (people who smoke: 15.1% had a college diploma; SFP users: 23% had a college diploma). However, SFP users had a higher mean monthly income (3,010 € vs. 2,560 €).

**Table 1:** Sociodemographic characteristics of the study population SFP: smoke-free product

Characteristic	People who smoke (n=1,000)	SFP users (n=196)
Gender		
Male	57.2%	50.0%
Female	42.8%	49.5%
Other	-	0.5%
Age (y)		
19-34	26.7%	23.5%
35-49	25.4%	38.3%
50-64	36.4%	33.7%
≥65	11.5%	4.6%
Mean age (y)		
	47.3	45.1
Monthly net income (€)		
<750	4.2%	2.0%
750-999	6.9%	1.0%
1,000-1,499	13.1%	9.2%
1,500-1,999	14.2%	10.2%
2,000-2,999	24.0%	26.5%
3,000-3,999	15.5%	26.5%
≥4,000	14.4%	18.4%
Not answered	7.7%	6.1%
Mean income (€)		
	2560	3010
Employment status		
Student	2.3%	1.5%
House wife/husband	4.6%	6.1%
Retired	21.1%	9.7%
Part-time	13.1%	15.3%
Full-time	49.5%	58.7%
Study	8.7%	8.2%
Highest educational level		
Not answered	1.4%	-
College with degree	15.1%	23.0%
College without degree	3.0%	3.6%
High school diploma	14.6%	16.3%
High school w/o diploma	40.4%	40.3%
Elementary with apprenticeship	21.1%	14.8%
Elementary w/o apprenticeship	4.5%	2.0%
Other adult smokers in the household		
Yes	41.5%	37.8%
No	58.5%	62.2%

More than half (58.9%) of the participants who smoked had done so for >20 years, with an average consumption of 16 cigarettes/day (Table 2). As defined by the study inclusion criteria, all SFP users had smoked cigarettes before switching, of which 87.8% smoked daily and 12.2% smoked occasionally. Before switching to SFPs 43.4% smoked for >20 years. Fifty-three participants still smoked a cigarette occasionally (five to 14 times/month) or rarely (three to four times/month), while 143 reported very rarely (one to two times/month) or never smoking. As specified in the study design, none of the SFP users reported smoking ≥15 times per month. To better separate the samples of people who smoke and use SFPs, participants who reported regular smoking (≥15 cigarettes/month) were excluded. In this sample, only users of E-cigs/HTPs who had switched from cigarettes and still smoked cigarettes “occasionally” at most (<15 times/month) were surveyed.

**Table 2:** Self-reported current and past use of TNPs SFP: smoke-free product; E-cigs: e-cigarettes; HTP: heated tobacco product; TNP: tobacco and nicotine product

Question	People who smoke (n=1,000)	SFP users (n=196)	E-cig (n=127)	HTP (n=69)
Smoking history				
Have you smoked before? (SFP users)				
Yes, daily	-	87.8	89.8	84.1
Yes, occasionally	-	12.2	10.2	15.9
How long have you smoked?				
<12 months	0.2	5.1	5.5	4.3
1-2 years	2.4	2.6	2.4	2.9
3-5 years	4.4	10.2	9.4	11.6
6-10 years	12.2	15.3	15.0	15.9
11-20 years	21.0	22.4	17.3	31.9
>20 years	58.9	43.4	48.8	33.3
Do not know/no answer	0.9	1.0	1.6	0
Which of these products have you used in the past seven days?				
Factory-made cigarettes	81.0	8.7	6.3	13.0
Roll-your-own cigarettes	48.7	5.6	2.4	11.6
E-cigs	0	73.5	100.0	24.6
HTPs	0	35.2	0	100.0
Snus/oral nicotine pouches	1.5	3.6	1.6	7.2
None of these	0	0	0	0
On average, how many cigarettes do you smoke per day?				
Daily				
1-2 cigarettes	2.8	0	0	0
3-5 cigarettes	9.4	0	0	0
6-10 cigarettes	24.6	0	0	0
11-20 cigarettes	46.6	0	0	0
21-50 cigarettes	16.2	0	0	0
>50 cigarettes	0.4	0	0	0
Average daily consumption	16.0	0	0	0
I do not smoke daily, but				
Often (15-29 times/month)	0	0	0	0
Occasionally (5-14 times/month)	0	15.3	12.6	20.3
Rarely (3-4 times/month)	0	11.7	11.8	11.6
Very rarely (1-2 times/month)	0	16.8	17.3	15.9
Never	0	56.1	58.3	52.2
On average, how often do you use your SFP?				
Daily	-	88.9	89.8	82.4
15-29 days/month	-	11.1	10.2	17.6
<15 days/month	-	0	0	0
On average, how many heated tobacco sticks do you consume?				
Daily				
1-2 sticks/day	-	4.3	-	4.3
3-5 sticks/day	-	8.7	-	8.7
6-10 sticks/day	-	27.5	-	27.5
11-20 sticks/day	-	43.5	-	43.5
21-50 sticks/day	-	8.7	-	8.7
>51 sticks/day	-	0	-	0
Not daily, but 15-29 days/month	-	7.2	-	7.2

Among E-cig users, 89.8% reported using the device daily, and 10.2% used it 15-29 days/month. The majority (77.2%) only used E-cigs with nicotine, 10.2% only used E-cigs without nicotine, and 12.6% used both types. Among HTP users, 82.48% reported using the device daily, and 17.6% used it on 15-29 days/month. Regarding daily consumption, 4.3% of HTP users reported using one to two tobacco sticks/day, 8.7% reported using three to five sticks/day, 27.5% reported using six to 10 sticks/day, 43.5% reported using 11-20 sticks/day, and 8.7% reported using 21-50 sticks/day. Of all SFP users, 15.3% smoked occasionally (five to 14 days/month), 11.7% rarely (three to four times/month), 16.8% very rarely (one to two times/month), and 56.1% never (Figure 5 of appendices).

Overall motivation to quit smoking and quit attempts among people who smoke

**Figure 1:**
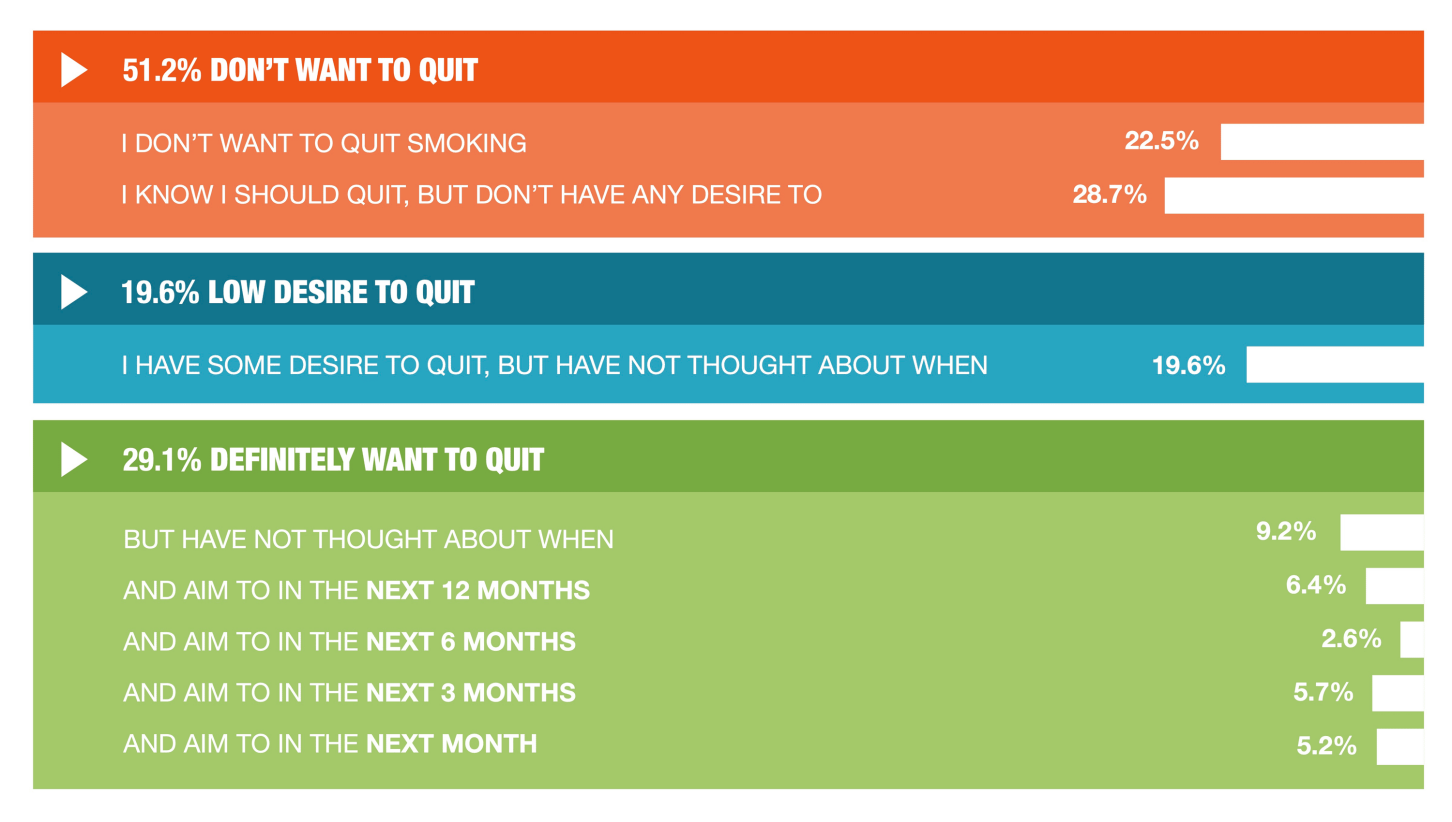
Motivation to quit smoking among adults who smoke (n=1,000) in Germany Participants were asked to identify one of eight statements that best applied to them regarding quitting smoking cigarettes.

People who smoke who currently lack the motivation to stop smoking were captured in the modified MTSS tool by the response categories one (“I do not want to stop smoking”) and two (“I think I should stop smoking but do not really want to”) [32,33]. The majority (51.2%) of 1,000 participants who smoked were not motivated to quit, with 22.5% not wanting to quit, and a further 28.7% knowing they “should quit,” but having no desire to stop (Figure 1). Another one-fifth (19.6%) had some desire to quit but had not decided when to try. The remaining 29.1% stated a strong desire to quit smoking at some point in the future (Figure 1).

Age

Older people who smoke showed particularly low motivation to quit. For example, among 50 to 64-year-olds, 56.4% had no desire to quit, rising to 64.4% among those aged 65+. In comparison, only 38.9% of participants aged 19-34 had no desire to quit. Older people who smoke (50-64 years) also reported the highest average daily consumption (17 cigarettes/day), which was more than that in the other three age groups (14-15 cigarettes/day) (Table 3).

**Table 3:** Motivation to quit smoking by age group among adults who smoke in Germany (n=1,000)

	19-34 y (n=195)	35-49 y (n=276)	50-64 y (n=398)	≥65 y (n=131)
Average daily cigarette consumption	14	15	17	15
No. of previous quit attempts				
In total	2.6	2.5	2.6	2.3
Last 12 months	1.3	1.0	1.0	0.9
Attitude toward quitting				
I do not want to quit	13.8%	25.1%	24.0%	32.7%
I know I should quit but do not have any desire to	25.1%	25.9%	32.4%	31.7%
I have some desire to quit but have not thought about when	27.5%	17.9%	16.5%	14.9%
I definitely want to but have not thought about when	8.7%	11.0%	9.4%	6.1%
I definitely want to quit and aim to				
Within 12 months	7.1%	8.8%	5.0%	3.7%
Within 6 months	1.9%	4.7%^ c^	1.7%	2.2%
Within 3 months	7.9%	3.7%	5.8%	4.6%
Within 1 month	8.0%	2.9%	5.2%	3.7%

Socioeconomic Status

**Figure 2:**
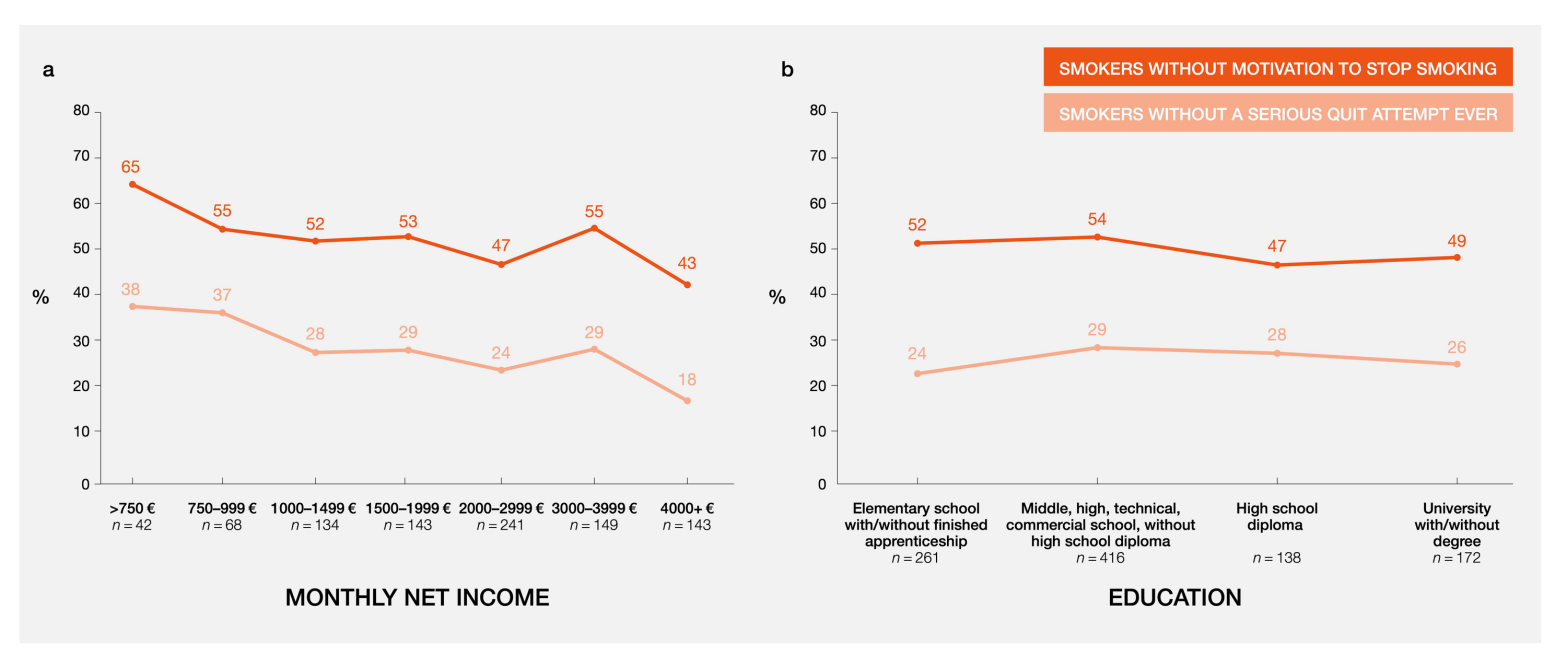
Lower motivation to quit smoking and fewer serious quit attempts are associated with lower socioeconomic status: a) influence of income; b) influence of education Shown are the percentages of people who smoke selecting the responses “I do not want to quit smoking” or “I know I should quit, but I do not have any desire to,” as well as the percentage of those answering “None” to the question: “How many serious attempts to quit smoking cigarettes have you made in total?”

Respondents with lower socioeconomic status had lower motivation to quit in comparison to respondents with a higher socioeconomic status (Figure 2), which was particularly noticeable with respect to monthly net income.

Quit Attempts

People who smoked were asked about the number of serious quit attempts they had made. When questioned about the previous 12 months, 53.9% of all people who smoke reported no attempts, 18.6% reported one serious attempt, and 19.4% reported two or three serious attempts to quit (Table 4). The mean number of serious quit attempts among all people who smoke in the past 12 months was 1.1. Consistent with the above findings on motivation to quit, the proportion of people who smoke without a serious quit attempt in the past 12 months was much higher among older people who smoke (60.5%-60.9%, age ≥50 years) than among the youngest people who smoke (43.1%, age 19-34 years) (Table 8 of appendices).

**Table 4:** The number of serious attempts to quit smoking among adults who currently exclusively smoke and among SFP users (i.e., ex-smokers) when they were regular smokers SFP: smoke-free product

Number of quit attempts	People who smoke		SFP users
	In the past 12 months	Ever	Ever
0	53.9%	26.9%	25.0%
1	18.6%	12.9%	15.8%
2-3	19.4%	35.1%	34.7%
4-5	3.8%	11.7%	12.8%
6-7	1.2%	5.5%	5.6%
8-9	0.2%	1.3%	0.5%
10 or more	0.8%	3.9%	3.6%
Do not know/no answer	2.1%	2.8%	2.0%
Mean number	1.1	2.5	2.5

Roughly a quarter (26.9%) of people who smoke had never attempted to quit after they had started smoking (Table 4). The proportion of people who smoke without a serious quit attempt was higher among older people who smoke (31.8%-32.4%, age ≥50 years) than among the youngest people who smoke (21.0%, age 19-34 years). The mean number of total quit attempts undertaken by the participants who smoked was 2.5. SFP users were also asked about the total number of serious quit attempts they had made during the time they exclusively smoked cigarettes. When comparing total previous serious quit attempts, similar proportions of people who smoke and SFP users had undertaken 0, 1, or 2-3 quit attempts (Table 4), and the mean number of total quit attempts made was also 2.5.

In summary, this study found that older people who smoke and those with lower socioeconomic status are less motivated to stop smoking and have had fewer attempts at quitting smoking as compared with younger people who smoke and those with higher education and incomes.

Barriers to quitting smoking

**Figure 3:**
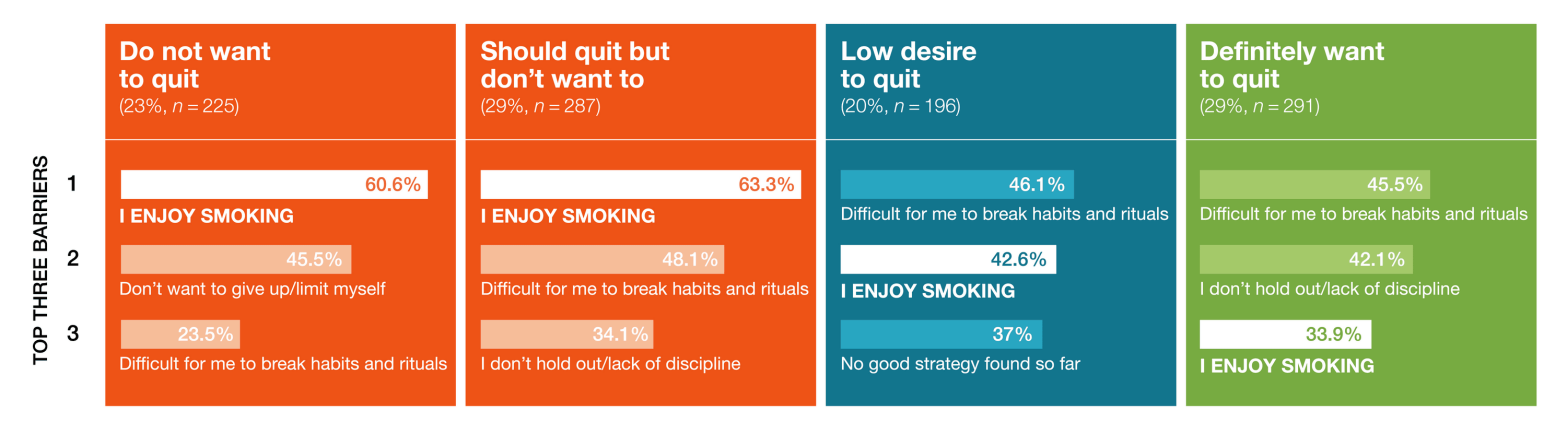
The top three barriers to quitting, as reported by adults who currently smoke, stratified by their degree of quitting motivation The barrier “enjoyment of smoking” featured in the top three barriers at all four levels of quitting motivation.

The closed question “From your point of view, what are crucial obstacles and difficulties that prevent you from quitting smoking cigarettes?” posed to people who currently smoke most frequently elicited the response “enjoyment of smoking” (50.1%), followed by “difficulties in breaking habits and rituals” (41.4%), and “lack of discipline” (31.2%) (Table 9 of appendices). Barriers to quitting smoking were further stratified by motivation to quit. “Enjoyment of smoking” was a particularly prevalent barrier chosen by the 51.2% who reported “no motivation to quit” (61%-64%) but was less of a barrier in those who “definitely wanted to quit” (33.9%, Figure 3). People who smoke and “wanted to quit” were more likely to mention “difficulties in breaking habits and rituals” (46.1%) as a barrier to quitting.

Among SFP users, when considering the time when they were exclusive smokers, the top three responses to the closed question were the same as in the cigarette smokers, “enjoyment of smoking” (51.5%), followed by “lack of discipline” (45.9%), and “difficulties in breaking habits and rituals” (44.4%).

Barriers to quitting smoking were also explored with the open question: “Why have not you quit smoking cigarettes yet?/What prevents you from doing so?,” whereas SFP users were asked to consider what had prevented them from quitting when they were exclusively smoking. When answers were grouped by theme, the most frequent response among people who smoke were “not having the will/not being ready to do it” (21.8%), followed by “enjoyment of smoking/liking/not wanting to quit” (18.0%), “nicotine addiction/dependence” (16.1%), and “stress” (13.4%) (Table 10 of appendices).

When considering barriers by age group, a high percentage of people who smoke of all ages reported “not having the will/not being ready to do it,” “enjoyment of smoking/liking/not wanting to quit,” and “nicotine addiction/dependence”; however, there were notable differences in other barriers (Table 5). For example, “stress” and “too many people who smoke in vicinity/partner or colleagues smoke” were frequent barriers for younger people who smoke (20.5% and 12.4%, respectively) but not for those aged 65 years and older (2.7% and 1.6%, respectively); in contrast, “enjoyment of tobacco/like the taste/tastes good” was a more common barrier for older people who smoke (12.3%) than for younger people who smoke (7.1%). Despite these differences in perceived barriers, younger people who smoke were more motivated to quit (Table 3).

**Table 5:** Barriers to quitting smoking among adults who smoke by age group (open question)

Response	Percentage of 19-34 y (n=195)	Percentage of 35-49 y (n=276)	Percentage of 50-64 y (n=398)	Percentage of ≥65 y (n=131)
The will is missing/not ready for it/can not do it	22.1	22.3	22.4	18.5
I enjoy smoking/I like it/I do not want to stop	18.6	19.5	17.8	14.3
The addiction/nicotine addiction/craving too big/dependence	14.6	16.2	17.2	16.2
Stress/work-related stress/everyday stress	20.5	16.4	9.5	2.7
Habit/I need to	17.3	9.0	13.2	8.9
Enjoyment of tobacco/like the taste/tastes good	7.1	5.3	9.8	12.3
Calms the nerves/helps me become calmer, more relaxed	8.6	8.5	6.2	2.1
Too many smokers in the vicinity/partner or colleagues smoke	12.4	6.3	3.5	1.6
Relapsed again and again/tried several times	2.2	4.4	2.1	3.2
Fear of weight gain	1.9	4.4	1.8	2.2

Lastly, the people who smoke were asked what would motivate them to quit, and the SFP users what had motivated them to quit cigarette smoking. “Reducing health risks” was the most frequent response in both people who smoke (73.3%) and SFP users (74.0%), followed by “saving money” (61.2% and 57.7%) and “not wanting to be addicted to smoking any more” (38.7% and 42.3%; data not shown).

Overall, when considering both questions, “enjoyment of smoking” was the most-cited barrier to smoking cessation, particularly among people who smoke who were not motivated to quit.

Perceptions of health risks

**Figure 4:**
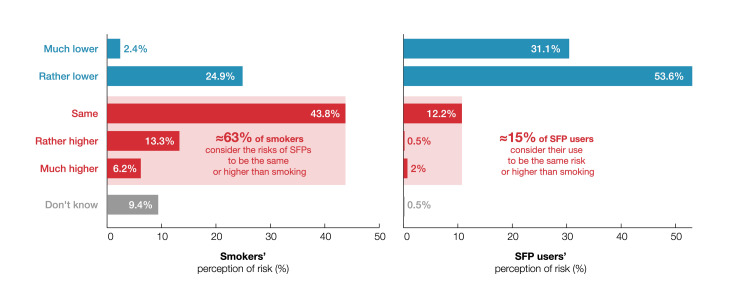
Relative risk of using SFPs versus smoking cigarettes, as perceived by adults who currently exclusively smoke (left) and by SFP users who are former exclusive smokers (right) SFP: smoke-free product

**Figure 5:**
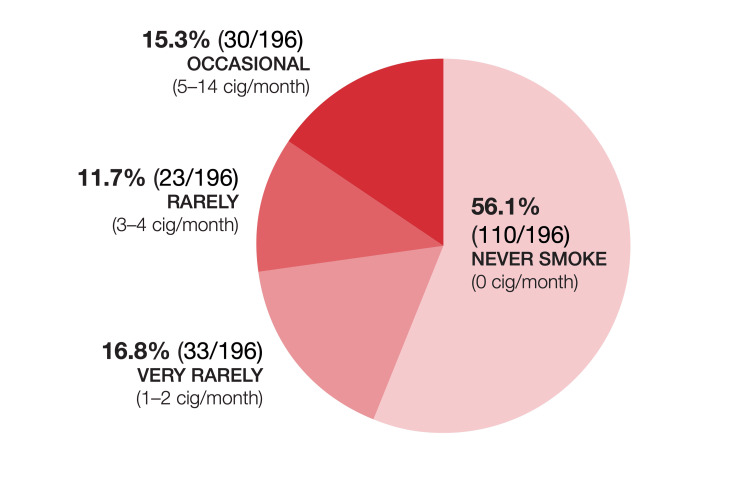
Cigarette consumption among 196 SFP users SFP: smoke-free product

All participants were asked about their perceptions of the health risks of SFPs relative to cigarettes (Figure 4) with the question: “How do you judge the health risk of E-cigs and HTPs compared to cigarettes?” Among people who smoke, 63.3% estimated the health risks of SFPs to be the same or greater than that of cigarettes versus just 14.8% of SFP users. Nearly 10% of the people who smoke were undecided, compared with under 1% of SFP users.

The people who smoke were asked “What has kept you from using E-cig/HTPs so far?” with respondents able to select more than one response. The most frequent responses given were “Not an adequate substitute for cigarettes" (40.6%), “I do not like the taste” (28.3%), and “Costs are too high” (25.7%), followed by “I am uncertain about harm potential” (23.0%), “Lack of information” (19.7%), and “Too much technology” (14.9%).

All participants were asked, “Which of the following substances in cigarette smoke is the responsible cause for typical smoking-associated diseases?” Among the people who smoke, 18% perceived the combustion of tobacco as the primary cause of the harmfulness of cigarette smoke versus 38.8% of SFP users. Nicotine was seen by 11% of people who smoke and 5.6% of SFP users as the primary cause of the harmfulness of cigarette smoke. Nicotine and the combustion of tobacco were seen as equally responsible for the harmfulness of cigarette smoke by 65.6% of people who smoke and 53.6% of SFP users (Table 6).

**Table 6:** Substance(s) in cigarette smoke primarily causing smoking-related diseases, as perceived by adults who currently exclusively smoke and by SFP users who are former exclusive smokers SFP: smoke-free product

Perceived primary cause of harmfulness of cigarette smoke	People who smoke (n=1,000)	SFP users (n=196)
Nicotine	11.7	5.6
Combustion products of tobacco (polycyclic hydrocarbons, carbon monoxide, etc.)	18.0	38.8
Nicotine and combustion products of tobacco	65.6	53.6
Do not know/no answer	4.8	2.0

Dialogue with physicians

Physicians play an important role in promoting healthy choices and encouraging people who smoke to quit; therefore, the study population was asked whether they had ever had a conversation with their doctor about quitting. The majority of people surveyed who smoke are not having these important conversations with their doctors. In fact, 51.4% replied “Never” to the statement “My doctor brings up this topic with me,” while 68.7% replied “Never” to the statement “I talk to my doctor about this topic,” and 78.7% replied “Never” to the statement “A conversation with my doctor has already led to an attempt to quit smoking”. The proportions were similar among SFP users (Table 11 of appendices).

Past dual use among current SFP users

To understand how the current SFP users had successfully switched from smoking cigarettes, we asked them about the transition period. While ongoing dual use was excluded by inclusion criteria, 82 (41.8%) of the 196 SFP users reported experiencing a phase of dual use before giving up smoking completely.

The most frequently reported dual-use duration was less than one month (30.5%), and most (89.1%) participants reported dual use for less than one year (Table 7). Among the 82 former dual users, 96.3% reported that they reduced their cigarette consumption during this period.

**Table 7:** Duration of past dual use phase among SFP users SFP: smoke-free product

Duration	Percentage of SFP users (n=82)
<1 month	30.5
1-2 months	24.4
3-6 months	28.0
7-12 months	6.1
>12 months	8.5
Do not know/no answer	2.4

## Discussion

This survey builds on the 2021 study, investigating the possible factors contributing to the stagnated smoking prevalence rate in Germany [28]. This study finds low motivation to quit and further reports the associated sociodemographic factors and main barriers that people who smoke face in trying to quit. Our findings may be applicable to other European populations, as a recent two-wave study across six European countries concluded that the factors influencing decisions about quitting may be shared among countries [34].

Similarly to the 2021 study, just over half of people who smoke reported no motivation to stop smoking, with 22.5% not wanting to quit, and 28.7% having no desire to quit although they thought they “should quit.” Among the 29.1% of people who smoke who want to quit smoking cigarettes in the coming year, quit plans were rarely specific, with just 5% planning to quit in the coming month. This finding is consistent with the DEBRA study for the period 2016-2019, which reported that 61.1% of people who smoke did not want to quit [32], as well as an analysis of DEBRA study data from 2016-2021, which found that more than 75% of people who smoke reported no motivation to quit [35].

To understand this lack of motivation, responses were correlated with different sociodemographic characteristics. People who smoke in the lowest income group were twice as likely as those in the highest income group to have never attempted quitting (38% vs. 18%). This group also had a higher proportion of smokers lacking motivation to quit (65% vs. 43%). A similar, although less obvious, pattern was seen with educational attainment.

Enjoyment of smoking, habits and rituals, and lack of discipline were the most frequently cited quit barriers. Specifically, “I enjoy smoking” was the top barrier to quitting for more than half of the people who smoke surveyed. Moreover, it is the primary barrier among more than 60% of people who smoke who lack motivation to quit smoking. People who smoke and are both unmotivated to quit smoking and cite the barrier “I enjoy smoking” are unlikely to seek the quit advice described in medical guidelines. In addition to measures boosting motivation to quit, this large group of people who smoke might benefit from information about tobacco harm reduction.

Remarkably, when SFP users reflected on the period when they were smoking regularly, they reported very similar quit barriers. Further, it was observed that even long-standing people who smoke can succeed, as 43.4% of the SFP users had smoked for more than 20 years before switching. It is important to learn how SFP users manage to switch, thus it should be explored in future surveys. Taken together, there are clear indications that switching to SFPs, as a concept of tobacco harm reduction implies, could be effective for achieving abstinence from cigarette smoking, especially for people who smoke who do not want to quit and cite “I enjoy smoking” as their biggest quit barrier.

In the UK, where public health bodies endorse the concept of tobacco harm reduction, success rates for achieving abstinence from cigarette smoking with E-cigs are comparable to or higher than with NRTs alone [36,37]. A naturalistic randomized controlled trial in the US examined what happens when people who smoke across the motivational spectrum are offered E-cigs with minimal instructions (and no cessation-focused support). The results indicate that unguided E-cig use leads to smoking abstinence, even in those who initially expressed little interest in quitting [38]. Despite these encouraging observations, the acceptance of SFPs in Germany remains low, perhaps due to widespread misperceptions about nicotine. In a 2019 survey by the German Federal Institute for Risk Assessment (BfR), 61% of people who smoke thought that the health risks of using E-cigs were equal to or higher than smoking cigarettes [20]. Similarly, this study found that over 60% of people who smoke thought that the health risks of SFPs are the same or higher than those of cigarettes. In contrast, nearly 85% of SFP users believed that these products are less risky than cigarettes, which is in line with the assessment by some public health bodies, including the UK Office of Health Improvement and Disparities, the UK’s Royal College of Physicians, and the German Cancer Research Center [9,19,39]. As highlighted in a 2024 publication on data from 11 waves of DEBRA, educational campaigns could help current smokers understand the comparative health risks of cigarettes and SFPs [40].

This widespread misconception about nicotine might act as a deterrent that prevents people who smoke from trying SFPs or NRTs [15,41,42]. Although NRTs are widely available in Germany, only 26% of people who smoke and 33% of SFP users in the survey had tried them (data not shown).

The need for accurate information and targeted education is underscored by the results of the survey. Two of the most common responses from people who smoke about why they have not tried E-cigs/HTPs are “uncertainty about potential harm” and “lack of information.” These sorts of misperceptions are seen in other countries, even in the UK, where E-cigs are more widely used. An annual survey by Action on Smoking and Health (ASH.org) indicates that the confusion about the health risks of E-cigs is increasing year on year [41].

Debate continues about whether dual use is a positive or negative phenomenon. Our survey cannot answer this question conclusively since inclusion criteria predominantly targeted people who successfully transitioned from cigarettes to SFPs. Of the SFP users in the survey, 42% reported a period of past dual use before switching completely. For more than half (55%) of these dual users, this period was short (six months or less). Similar observations have been made in other countries, such as the UK and US [38,43]. However, some have raised concerns that dual use might be equally or more harmful than exclusive smoking [44-46]. Clearly, dual use is not an ideal scenario from a harm reduction perspective. However, several studies have begun to examine the biological impact of dual use, finding that it generally reduces the number of cigarettes smoked per day, and confers a reduction in biomarkers of harm [47,48]. Rüther et al. observed that people who smoke, when given E-cigs and usage advice, substituted cigarettes with E-cigs by up to 70% and showed improvements in lung health [49]. Furthermore, dual use can be a stepping stone to abstinence [38,43]. This might be partially attributable to dual use providing a way for people who smoke to start changing their habits and behaviors, which was one of the top three barriers to quitting among unmotivated people who smoke in our study.

Physicians are regarded as an important source of health-related information for people who smoke in Germany [29]. As such, they should play an important role in promoting cessation. For those who would otherwise continue to smoke, physicians should be able to convey accurate information about tobacco harm reduction. In more than half of the people who smoke surveyed here, the subject of smoking had not been brought up with their physician, and nearly 70% of respondents said they had never discussed it. These results suggest that physicians in Germany could be more active in encouraging people who smoke to quit.

Smoking cessation is not a standard part of medical training in Germany [50]. A randomized controlled trial in German primary care evaluated whether such training would be impactful [51,52]. A follow-up analysis showed that patients who received medical advice were much more likely to attempt quitting and/or achieve abstinence at a six-month follow-up [53]. However, results suggest these measures have not found their way into daily medical practice. In contrast, in the US and UK, more than half of people who smoke receive advice from their physician, which has increased the number of quit attempts [54,55].

Science-based education of healthcare professionals and impartial consideration of scientifically substantiated SFPs and tobacco harm reduction might, therefore, complement existing measures to reduce the detrimental effects of smoking [56]. Physicians worldwide could benefit their patients by learning how to correct misinformation about nicotine and SFPs [22,23,25].

The E-cig is the most commonly used product to abandon cigarette smoking in Germany, and it is possible that people who smoke could decide to use HTPs in a similar way [3]. Nicotine delivery is an important factor in determining whether people who smoke find an SFP acceptable, and both E-cigs and HTPs sufficiently deliver nicotine [57-59].

Interestingly, both Germans of advanced age and those with lower socioeconomic status have relatively low health literacy [60]. Whether or not this correlates with smoking parameters should be investigated further. Meanwhile, sociodemographic and socioeconomic insights should be factored into prevention measures and future campaigns to encourage smoking cessation.

This study has several strengths. First, the study recruited a large number of people who smoke via two consumer panels. Although the survey only included 196 SFP users, it does allow us to start to contextualize and compare this group with people who smoke. Second, the survey population was representative of the people who smoke cigarettes exclusively, employing recruitment quotas on age, sex, and German state of residence. Third, the measure of motivation to stop smoking was derived from the previous MTSS [32,33]. Fourth, this is the first study to explore barriers to smoking cessation in Germany, thus complementing the DEBRA study. Fifth, the use of both a closed and an open question to probe barriers to quitting provides a more reliable view of the study population. Our conclusions are strengthened by the fact that both questions identified “enjoyment of smoking” as a key barrier. Finally, the dataset can be easily compared with the 2021 dataset [28].

There are some limitations to this study. The use of quota sampling and a non-probability sample limits the generalizability of the results. Since the study only surveyed people living in Germany, it is unclear how these barriers vary across different cultural contexts. Because individuals who used SFPs and smoked more than 14 cigarettes per month were excluded, this sample is not representative of all dual users of E-cigs/HTPs in Germany. This limitation draws attention to the fact that many surveys do not ask sufficiently nuanced questions about product use. More accurate use of information is needed to understand how smokers can overcome barriers to quitting.

## Conclusions

The best way to reduce the health risks of smoking is to quit smoking altogether. However, focusing solely on cessation does not acknowledge the real challenges that people who smoke (barriers to quitting) face when trying to quit. The survey results, specifically on the lack of motivation to quit, suggest that current tobacco control measures are not effectively motivating the majority of people who smoke in Germany to quit. This study demonstrated that half of the people who smoke surveyed are not motivated to quit, and only 5% plan to quit in the next month. Older segments of the study population (aged 50+) and those in lower-income brackets are even less motivated to quit, with smoking enjoyment being the biggest barrier to quitting, affecting more than 60% of people who smoke and do not want to quit.

SFP users were more likely to perceive the risks of SFPs and causes of smoking-related disease more in line with the current scientific evidence than people who smoked cigarettes. Lack of motivation to quit smoking and barriers to quitting manifest in different ways, implying that differentiated approaches are required to help people who smoke successfully move away from smoking cigarettes. They should have access to accurate information on the role of combustion-generated toxicants as the primary cause of smoking-related diseases and the relative risks of SFPs compared with continuing to smoke. Sustainably reducing smoking prevalence in Germany will require an integrated strategy that complements the existing tobacco control and prevention measures with tools based on the principles of tobacco harm reduction.

## Appendices

**Table 8:** Quit attempts among people who smoke: percentage of attempts to quit smoking in the past 12 months and ever, by age group, among 1,000 people who smoke

No. of attempts	19-34 y (%)	35-49 y (%)	50-64 y (%)	≥65 y (%)
In the past 12 months				
0	43.1	52.6	60.5	60.9
1	23.6	21.3	14.7	13.6
2-3	23.4	17.7	18.5	16.5
4-5	5.6	3.0	3.4	3.0
6-7	1.8	1.1	1.2	-
8-9	-	0.4	0.2	-
≥10	1.0	0.4	0.7	1.7
Do not know/no answer	1.6	3.4	0.8	4.4
Ever				
0	21.0	23.0	32.4	31.8
1	16.8	14.2	10.3	9.4
2-3	36.2	37.4	32.2	36.5
4-5	13.9	10.1	11.6	10.2
6-7	4.3	7.8	4.7	5.3
8-9	1.5	0.8	1.6	0.8
≥10	3.6	2.7	5.3	2.5
Do not know/no answer	2.5	4.0	1.8	3.5

**Table 9:** Barriers to quitting smoking identified through the closed question The percentages of people who smoke and SFP users’ responses to the closed question: “From your point of view, what are/were the crucial obstacles and difficulties that prevent/-ed you from quitting smoking cigarettes?” The responses were grouped into five categories: capability, opportunity, attitude, social support, and self-efficacy. SFP: smoke-free product

Barrier	% of people who smoke (n=1,000)	% of SFP users (n=196)
Capability		
Do not know well enough about methods/tools	6.8	6.6
I do not handle withdrawal symptoms well	17.1	23.0
I have not found a good strategy yet	24.3	21.4
Opportunity		
Do not know where to find support	4.5	2.0
Supporting offers and products are too expensive or me	12.1	10.2
Attitude		
General possible withdrawal symptoms	19.3	22.4
Does not fit into my current daily routine	21.9	25.5
Do not want to give up smoking	21.2	34.2
Less downtime, breaks, or moments for me	15.8	15.8
I enjoy smoking	50.1	51.5
Social support		
No/barely any support from friends, family, and acquaintances	6.5	3.6
Many smoking people in my environment	28.6	37.8
Limitation of social moments with other smokers	23.6	36.7
Self-efficacy		
Unsuccessful trials so far/worried about failing again	13.7	10.2
I do not hold out/lack discipline	31.2	45.9
It is difficult for me to break habits and rituals	41.4	44.4
I have lost the motivation and have given up trying to quit	8.5	20.9

**Table 10:** Barriers to quitting smoking identified through the open question The percentages of people who smoke and SFP users’ responses to the open question, “Why have not you quit smoking cigarettes yet?/What prevents you from doing so? Please describe your answer as detailed as possible.” SFP: smoke-free product

Response	% of people who smoke (n=1,000)	% of SFP users (n=196)
The will is missing/not ready for it/can not do it	21.8	10.2
I enjoy smoking/I like it/I do not want to stop	18.0	4.6
The addiction/nicotine addiction/craving too big/dependence	16.1	16.3
Stress/work-related stress/everyday stress	13.4	4.1
Habit/I need to	12.8	10.7
Enjoyment of tobacco/like the taste/tastes good	8.2	3.1
Calms the nerves/helps me become calmer, and more relaxed	7.0	2.6
Too many smokers in the vicinity/partner or colleagues smoke	6.4	7.1
Relapsed again and again/tried several times	2.9	1.0
Fear of weight gain	2.5	0.5

**Table 11:** Conversations with physicians: people who smoke and SFP users were asked to what extent they agreed with three statements

Question	% of people who smoke (n=1,000)	% of SFP users (n=196)
My doctor brings up this topic with me		
Yes, regularly	7.3	6.6
Yes, rarely	14.2	12.2
Yes, but occasionally	25.3	25.5
Never	51.4	53.1
Do not know/no answer	1.8	2.6
I talk to my doctor about this topic		
Yes, regularly	2.4	2.6
Yes, rarely	8.3	9.2
Yes, but occasionally	19.1	15.3
Never	68.7	71.9
Do not know/no answer	1.5	1.0
A conversation with my doctor has already led to an attempt to quit smoking		
Yes, regularly	2.5	2.6
Yes, rarely	6.0	4.6
Yes, but occasionally	10.2	12.2
Never	78.7	79.6
Do not know/no answer	2.6	1.0
